# Use of Sunscreen and Indoor Tanning Devices Among a Nationally Representative Sample of High School Students, 2001–2011

**DOI:** 10.5888/pcd11.140191

**Published:** 2014-08-21

**Authors:** Corey H. Basch, Charles E. Basch, Sonali Rajan, Kelly V. Ruggles

**Affiliations:** Author Affiliations: Charles E. Basch, Sonali Rajan, Teachers College, Columbia University, New York, New York; Kelly V. Ruggles, Center for Health Informatics and Bioinformatics, New York University Langone Medical Center, New York, New York.

## Abstract

Adolescents are particularly vulnerable to engaging in poor skin-protection behaviors. The objective of this study was to examine use of sunscreen and indoor tanning devices among a nationally representative sample of high school students during a 10-year period (2001–2011) using data from the Youth Risk Behavior Surveillance System. The percentage of youth who reported using sunscreen declined from 67.7% in 2001 to 56.1% in 2011. The prevalence of using indoor tanning devices was highest among white females: 37.4% in 2009 and 29.3% in 2011. These findings indicate the need for prevention efforts aimed at adolescents to reduce risks for skin cancer.

## Objective

Skin cancer is the most common cancer in the United States (1). From 2001 to 2010, incidence of melanoma increased annually by 1.6% among men and by 1.4% among women (2). Preventive measures, such as using sunscreen and not using artificial tanning devices, are recommended to avoid developing skin cancer (2,3). Skin-protection behaviors are especially important for children and adolescents because sun exposure during childhood and adolescence directly influences the development of skin cancer later in life (3). The objective of this study was to examine skin-protection behaviors among a nationally representative sample of high school students during a 10-year period (2001–2011) using data from the Centers for Disease Control and Prevention’s (CDC’s) Youth Risk Behavior Surveillance System (YRBSS).

## Methods

The CDC collects data through the YRBSS from a nationally representative sample of public, private, and parochial high schools every 2 years (4). The data represent each sex, race/ethnicity group, high school grade, and nearly every US state. Our study focused on the analysis of responses to 2 YRBSS questions: 1) When you are outside for more than one hour on a sunny day, how often do you wear sunscreen with an SPF of 15 or higher? and 2) During the past 12 months, how many times did you use an indoor tanning device, such as a sunlamp, sunbed, or tanning booth? The response format for each item has multiple categories and was subsequently dichotomized according to CDC methods (4). For the item on sunscreen use, the possible responses are “never,” “rarely,” “sometimes,” “most of the time,” and “always.” We dichotomized this variable into those who reported ever using sunscreen in the previous 12 months and those who reported never using sunscreen. For the item on indoor tanning devices, we dichotomized responses (0, 1 or 2, 3–9, 10–19, 20–39, and ≥40 times) into those who reported no use and those who reported any use; we further dichotomized the data into 1) used 1 to 19 times and 2) used more than 20 times (ie, heavy use). The item on sunscreen use was asked in the same way in all 6 years of survey administration studied (2001, 2003, 2005, 2007, 2009, and 2011). The item on use of tanning devices was asked only in 2009 and 2011.

We calculated for each survey year the percentage of youth who reported wearing sunscreen and the percentage of youth who reported using a tanning device. We calculated percentages by sex (male or female), race/ethnicity (Hispanic, black, white, and “other”), and grade level (9–12), and various combinations of subgroups. We excluded from analysis surveys that were missing data (<5% of surveys). All YRBSS data were weighted to match national population proportions (4). We created a heat map for data on sunscreen use, which allows readers to visualize fluctuations in prevalence. This study was deemed to be exempt by the institutional review board at William Paterson University and was approved by the institutional review board at Teachers College, Columbia University, and New York University Langone Medical Center.

## Results

The overall percentage of respondents who reported using sunscreen decreased from 67.7% in 2001 to 56.1% in 2011 (Figure). The lowest prevalence during the study period was in 2005 (55.3%). We observed similar patterns among white males and females. Overall, a greater percentage of females than males consistently reported using sunscreen. Similarly, white respondents consistently reported using sunscreen at higher rates than their black, Hispanic, and other racial/ethnic counterparts. Rates of sunscreen use were similar across grade levels.

**Figure:**
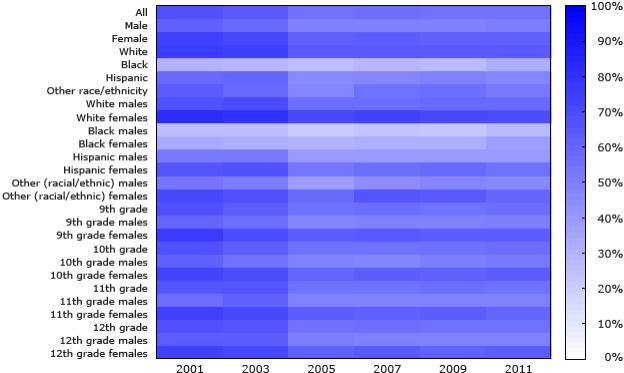
Prevalence of sunscreen use among a nationally representative sample of high school students, 2001–2011. Data source: Youth Risk Behavior Surveillance System. **Table:** 

Demographic group	2001	2003	2005	2007	2009	2011
	Percentage					
All	67.7	64.6	55.3	56.3	55.7	56.1
Male	61.1	59.2	48.8	49.0	49.7	50.4
Female	74.0	70.4	62.0	63.8	62.4	62.3
White	75.3	74.8	64.3	65.2	64.1	64.1
Black	29.7	29.2	25.4	28.2	27.2	32.4
Hispanic	58.8	60.2	46.5	48.2	49.5	47.3
Other racial/ethnic group	62.9	58.9	48.0	55.1	56.3	53.1
White male	68.7	69.7	57.5	57.0	58.1	58.8
White female	81.4	80.3	71.2	73.5	71.1	69.8
Black male	25.5	25.8	20.5	24.3	22.6	27.1
Black female	33.6	32.7	30.1	32.0	31.9	37.6
Hispanic male	51.9	51.8	40.0	39.9	40.6	39.9
Hispanic female	65.7	68.6	53.3	56.4	58.5	55.1
Other (racial/ethnic) male	54.0	51.4	37.5	44.8	47.6	45.6
Other (racial/ethnic) female	71.0	67.6	58.1	65.7	64.8	60.9
9th grade (all)	68.5	63.4	55.4	56.7	55.7	56.4
9th-grade male	60.8	57.5	47.5	49.2	49.1	50.2
9th-grade female	75.3	69.7	63.4	64.6	63.5	62.8
10th grade (all)	67.8	62.6	54.7	55.8	55.4	57.8
10th-grade male	62.4	55.6	49.0	47.9	50.3	52.8
10th-grade female	73.2	69.7	60.5	64.0	61.1	63.3
11th grade (all)	65.9	66.6	56.0	55.8	56.3	55.0
11th-grade male	57.7	61.9	49.3	49.4	49.6	49.7
11th-grade female	73.6	71.5	62.7	62.1	63.4	60.6
12th grade (all)	68.7	66.9	55.7	57.5	55.6	55.5
12th-grade male	63.8	63.2	50.1	49.6	49.9	48.9
12th-grade female	73.6	71.2	61.4	65.0	61.5	62.5

The percentage of respondents who reported using indoor tanning devices was 15.6% in 2009 and 13.3% in 2011 (Table). In 2009 and 2011, a greater percentage of female respondents (25.4% in 2009; 20.9% in 2011) than male respondents (6.7% in 2009; 6.2% in 2011) reported using indoor tanning devices. The use of indoor tanning devices was greatest among white females compared with all other subgroups: 37.4% in 2009 and 29.3% in 2011. The percentage of respondents who reported heavy use of indoor tanning devices in the previous 12 months was highest among white females (12.4% in 2009 and 11.0% in 2011). We also found that the prevalence of using a tanning device increased as grade level increased. 

**Table:** Prevalence of Using Indoor Tanning Devices Among a Nationally Representative Sample of High School Students, 2009 and 2011

Demographic group	2009	2011
**All**	15.6	13.3
**Sex**		
Male	6.7	6.2
Female	25.4	20.9
**Race/ethnicity**		
White	21.1	17.4
Black	4.5	3.9
Hispanic	8.2	7.6
Other	7.9	9.0
**Race/ethnicity and sex**		
White male	7.0	6.2
White female	37.4	29.3
Black male	6.1	4.5
Black female	2.7	3.3
Hispanic male	5.8	5.7
Hispanic female	10.6	9.6
Other (racial/ethnic) male	5.3	8.2
Other (racial/ethnic) female	10.3	9.8
**Grade level and sex**		
9th grade (all)	10.5	8.1
9th-grade male	5.9	4.5
9th-grade female	16.0	11.7
10th grade (all)	13.4	10.1
10th-grade male	4.6	4.9
10th-grade female	23.2	15.7
11th grade (all)	18.2	16.4
11th-grade male	7.0	6.8
11th-grade female	30.3	26.5
12th grade (all)	21.7	19.7
12th-grade male	10.0	8.5
12th-grade female	33.8	31.8

## Discussion

This is the first study to examine the most recent YRBSS data on skin-protection behavior across 10 years and multiple demographic subgroups. Other studies have examined YRBSS data on this topic, and their findings are similar to ours. A study in 2009 examined the number of high school students who used tanning devices during a 12-month period and found that although only 15.6% of students used a tanning device, nearly half of them did so 10 or more times (5). A study examining trends in sunscreen use during 1999–2009 found an increase in the percentage of high school students who never or rarely wore sunscreen on a sunny day for more than an hour (6).

A limitation to our study is that YRBSS data are cross-sectional; that data represent only certain points in time should be considered in the interpretation of prevalence patterns. Data are also based on self-report. Despite these limitations, the sample sizes are large, and the study fills a gap in knowledge. 

Overexposure to ultraviolet radiation is a risk factor for skin cancer (7). Prevention of overexposure is especially important during childhood and adolescence. Our research supports past findings that adolescents practice behaviors that increase the risk for skin cancer (8,9). Our findings also illustrate that certain demographic subgroups — girls and young women — are especially at risk for engaging in unsafe skin-protection practices; future preventive efforts should prioritize these young people.
